# Evolving Landscape of Long Non-coding RNAs in Cerebrospinal Fluid: A Key Role From Diagnosis to Therapy in Brain Tumors

**DOI:** 10.3389/fcell.2021.737670

**Published:** 2021-10-07

**Authors:** Kanghong Xu, Xinquan Jiang, Abakundana Nsenga Ariston Gabriel, Xiaomeng Li, Yunshan Wang, Shuo Xu

**Affiliations:** ^1^School of Public Health, Shandong First Medical University and Shandong Academy of Medical Sciences, Taian, China; ^2^Department of Clinical Laboratory, The Second Hospital of Shandong University, Jinan, China; ^3^Department of Hematology, Jining First People’s Hospital, Jining, China; ^4^Department of Neurosurgery, Qilu Hospital of Shandong University and Institute of Brain and Brain-Inspired Science, Shandong University, Jinan, China; ^5^Key Laboratory of Brain Function Remodeling, Qilu Hospital of Shandong University, Jinan, China

**Keywords:** lncRNA, CSF, brain tumor, biomarker, treatment, diagnostic

## Abstract

Long non-coding RNAs (lncRNAs) are a type of non-coding RNAs that act as molecular fingerprints and modulators of many pathophysiological processes, particularly in cancer. Specifically, lncRNAs can be involved in the pathogenesis and progression of brain tumors, affecting stemness/differentiation, replication, invasion, survival, DNA damage response, and chromatin dynamics. Furthermore, the aberrations in the expressions of these transcripts can promote treatment resistance, leading to tumor recurrence. The development of next-generation sequencing technologies and the creation of lncRNA-specific microarrays have boosted the study of lncRNA etiology. Cerebrospinal fluid (CSF) directly mirrors the biological fluid of biochemical processes in the brain. It can be enriched for small molecules, peptides, or proteins released by the neurons of the central nervous system (CNS) or immune cells. Therefore, strategies that identify and target CSF lncRNAs may be attractive as early diagnostic and therapeutic options. In this review, we have reviewed the studies on CSF lncRNAs in the context of brain tumor pathogenesis and progression and discuss their potential as biomarkers and therapeutic targets.

## Background

Brain tumors refer to the primary intracranial tumors as well as the metastatic tumors in the brain with a primary lesion. They account for 1.8% of newly diagnosed cancers and 2.3% of cancer-related deaths worldwide (Gerlinger et al., 2012; Xie et al., 2014; Hodges et al., 2017; Siegel et al., 2017). Patients often suffer from symptoms due to increased intracranial pressure (headache, nausea, and vomiting) and neurological dysfunction (seizures, hemiplegia, aphasia, and cognitive deficits) (Cahill et al., 2012; Hadidchi et al., 2019). The natural disease progression in these patients is characterized by a progressive neurological loss and a rapid decline in the quality of the patients’ lives.

While considerable advancements in fundamental scientific research and clinical practice have shed light on the brain tumor pathophysiology in the past decades, challenges remain in precise and early diagnosis (Sawaya et al., 1998; Wang and Bettegowda, 2017; Nevel et al., 2018). For instance, computed tomography (CT) (Lebanony et al., 2009) and magnetic resonance imaging (MRI) are reliable in determining the spatial and structural characteristics for brain tumors; however, these non-invasive approaches can hardly determine the pathological classification and malignant degree of tumors (Cao et al., 2017; Huang et al., 2019); Surgical biopsy can overcome these limitations by extracting brain tissue samples, while it is much more invasive, expensive, and risky. Added to the difficulties associated with accessing the nature of brain tumors, there are currently limited therapeutic options available nowadays, including (Galldiks et al., 2017; Laudicella et al., 2021) resection surgeries, chemotherapy, radiotherapy, and electric field therapy (Uddin et al., 2020; López Vázquez et al., 2021). Unfortunately, total removal of the target lesion cannot always be achieved while minimizing the risk of any postoperative functional decline attributable to the surgical procedure itself (Martinez-Rios et al., 2016; Duffau, 2017). Certain brain malignancies are also prone to drive tumor progression and resist chemo- and radiotherapy. Therefore, the outcome of patients with brain tumors is still miserable. The 5- and 10-year survival rates for central nervous system (CNS) tumors are about 36% and 31%, respectively. For the patients with newly diagnosed glioblastoma, the most malignant form of brain tumor, the median survival duration does not exceed 2 years (Martinez-Rios et al., 2016; Duffau, 2017; Jeon et al., 2021; Rankin-Turner et al., 2021; Reddy et al., 2021).

In this scenario, it is essential to develop new methodologies to determine the diagnostic and prognostic parameters for various brain tumors and guide the individualized therapy (Horbinski et al., 2019; Kristensen et al., 2019). Non-coding RNAs (ncRNAs), especially long non-coding RNAs (lncRNAs), have emerged as a novel family of master regulators, because they are widely involved in the development and progression of various CNS disorders, including brain tumors (Kang et al., 2011; Hawrylycz et al., 2012; Chen and Qin, 2015; Hanan et al., 2017). Also, ncRNA-based tumor liquid biopsy has been demonstrated recently at the preclinical level, which detects tumor-specific ncRNA in a less invasive manner from certain body fluids, including serum, urine, and cerebrospinal fluid (CSF). Compared to serum and urine, brain tumor associated ncRNAs are much enriched in the CSF. Therefore, CSF has been considered a promising candidate for brain liquid biopsy. In fact, dysregulation of CSF lncRNAs has been demonstrated in multiple tumors and non-tumor nervous system disorders, including Alzheimer’s disease (Zhuang et al., 2020), cerebral vasospasm secondary to subarachnoid haemorrhage (Pan et al., 2020), and cerebral ischemia-reperfusion injury (Zhang Y. et al., 2020). In this review, we focus on the regulatory mechanism of CSF lncRNAs in the pathophysiology of brain tumors and discuss its potential application as diagnostic markers and therapeutic targets.

## Functions and Characteristics of Cerebrospinal Fluid

The main limitation of blood biopsy for brain tumors is the low serum levels of tumor specific biomarkers, mainly caused by the brain blood barrier (BBB), which is formed by brain microvascular endothelial cells sealed by tight junctions (Banks, 2019). The BBB maintains the independent circulation of CSF and microenvironment homeostasis for the brain tissue (Sweeney et al., 2019). However, the BBB also brings significant challenges to diagnosis and treat brain tumors (Jeon et al., 2021; Pottoo et al., 2021). For instance, most tumor-specific antigens are confined within this barrier, and their systematic detection can be problematic. In addition, the poor BBB penetration naturally hinders the delivery of therapeutic drugs (such as chemotherapeutic drugs, targeted therapeutic drugs, and monoclonal antibodies) into the brain (Seo et al., 2020; Zhang et al., 2021). Although researchers have been developing strategies to modulate BBB permeability, most approaches are difficult to apply in the clinical setting (Sprowls et al., 2019; Pandit et al., 2020). Under these circumstances, CSF biopsy and CSF-based therapeutics have been gradually recognized as an alternative approach to overcome these obstacles.

**FIGURE 1:**
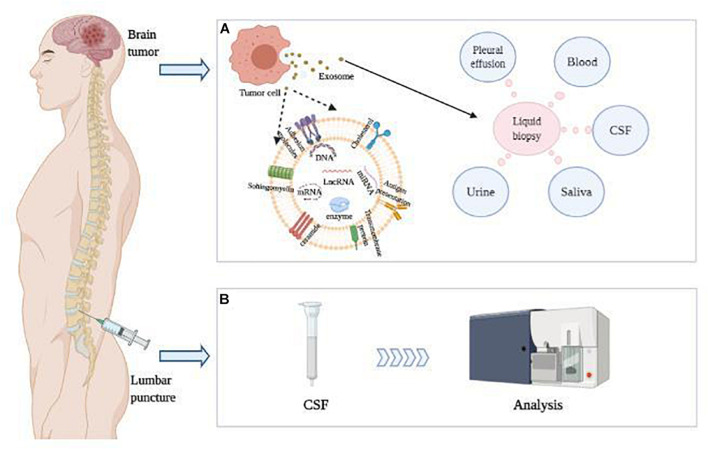
LncRNA in CSF were detected as diagnostic and prognostic indicators of brain tumors. **(A)** Brain tumor cells release exosomes containing nucleic acids, which can be further detected in various body fluids. **(B)** CSF was obtained by lumbar puncture, and CSF lncRNAs packaged in exosomes were detected as diagnostic and prognostic indicators of brain tumors.

The CSF is an ultrafiltrate of plasma surrounding the brain and spinal cord (Parnetti et al., 2019). The majority of CSF is produced by the choroid plexus, circulates through the ventricles, the cisterns, and the subarachnoid space to be absorbed into the blood by the arachnoid villi (Johanson et al., 2008). The CSF provides biological and mechanical support to the brain, transports nutrients, signaling molecules, and debris, and regulates brain immunity (Orešković, 2015; Tumani et al., 2017; Attier-Zmudka et al., 2019). Therefore, homeostasis in the production, circulation, and absorption of the CSF is critical for brain function (Proulx, 2021). Analogously, abnormal CSF mirrors the dysregulation of brain in various neurological diseases (Figure 1), mainly because CSF is more closely associated with small molecules, peptides, or proteins released from the brain tissues (Johanson et al., 2008; Sakka et al., 2011). Since CSF directly mirrors the biochemical processes in the brain, CSF components are widely used to identify pathogen invasion or diagnosis of neurological diseases (Figure 1). Lundborg et al. (2010) reported that glial cell line-derived neurotrophic factor (GDNF) was increased in the CSF of patients with long-term pain but decreased in the blood. Tumor-specific biomarkers are also enriched in the CSF. For instance, the levels of miR-10b and miR-21 are found significantly increased in the CSF of patients with glioblastoma and brain metastasis of breast and lung cancer, compared with tumors in remission and a variety of non-neoplastic conditions (Teplyuk et al., 2012). Similarly, significantly higher CSF levels of miR-21, miR-19b, and miR-92a were identified in primary CNS lymphoma (PCNSL) (Zajdel et al., 2019). Recently, it was reported that immune cell profiling of the CSF enabled the characterization of the brain metastasis microenvironment (Rubio-Perez et al., 2021).

## Long Non-Coding RNA in the Cerebrospinal Fluid

**FIGURE 2:**
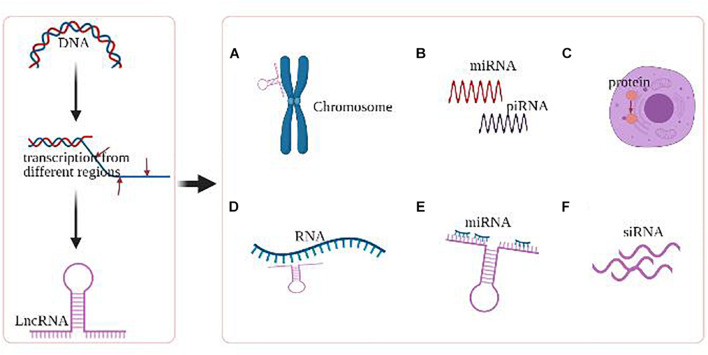
Major epigenetic regulations of lncRNA. **(A)** LncRNAs mediate chromosomal remodeling. **(B)** LncRNAs act as small RNA (miRNA, piRNA) precursor. **(C)** LncRNAs alter cellular localization of proteins. **(D)** LncRNAs involve in the transcriptional regulation of genes. **(E)** LncRNAs act as miRNA sponge. **(F)** LncRNAs produce endogenous siRNAs with the involvement of the dicer enzyme.

LncRNAs, of which length ≥ 200 bp, exhibit a wide range of regulatory activities based on their subcellular localization, including gene transcriptional regulation and mRNA splicing in the nucleus, as well as mRNA stability and protein function modulation in the cytoplasm (Figure 2; Ramanathan et al., 2019; Yao et al., 2019). Dysregulation of lncRNAs has been demonstrated to contribute to cancer development and progression *via* abnormal epigenetic alterations in oncogene regulation pathways., including abnormal DNA methylation or histone changes at their gene promoters. More specifically, emerging data suggest that lncRNAs comprise a network of epigenetic modulators by creating platforms for chromatin-remodeling complexes and transcription factors capable of modulating the transcriptional state of lncRNA-controlled genomic loci (Romani et al., 2018; Chen et al., 2021).

Like the other ncRNAs, lncRNAs are selectively packaged, secreted, and transferred between cells by exosomes, which are small bilipid layer enclosed extracellular nano-vesicles with various physiological and pathophysiological functions (Sullivan et al., 2017; Raposo and Stahl, 2019). With exosomes as vectors, lncRNAs are able to cross the BBB and readily accessible in CSF, making it an ideal diagnostic and therapeutic candidate for multiple diseases in the CNS, such as neurodegenerative disorders, stroke, multiple sclerosis, and brain tumors (Cheng et al., 2020; Rastogi et al., 2021). For instance, lncRNAs MALAT1 and SNHG4 are downregulated in the CSF samples of patients with Alzheimer’s disease and acute cerebral infarction, respectively (Zhang S. et al., 2020; Zhuang et al., 2020). lncRNA HIF1-AS3 transcriptomic downregulation in human choroid plexus tissue and abnormal PAI-1 level in CSF were observed in patients with progressive multiple sclerosis (Rodríguez-Lorenzo et al., 2020). Intriguingly, Pan et al. (2020) established a CSF lncRNA-based signature (ZFAS1, MALAT1, LINC00261, and LINC01619) to predictive cerebral vasospasm in patients with subarachnoid hemorrhage.

Abundant evidence has revealed that CSF lncRNAs were also extensively expressed in different brain tumors and involved in tumorigenesis, tumor progression, invasion, angiogenesis, and metastasis. In 2017, Ma et al. (2017) firstly observed that lncRNA HOTAIR derived from glioma cells promotes angiogenesis by regulating the endothelial VEGF expression. Similar to Ma’s observation, Bian et al. (2019) found that exosomal lncRNA-ATB triggered astrocytes to facilitate the glioma invasion. Moreover, Zhang et al. demonstrated that that lncRNA SBF2-AS1 was upregulated in chemotherapy-resistant glioblastoma (GBM), and overexpression of SBF2-AS1 led to the promotion of chemotherapy resistance, which was regulated by transcription factor ZEB1. ZEB1 directly binds to the SBF2-AS1 promoter region to regulate SBF2-AS1 level and affected temozolomide (TMZ) resistance in GBM cells (Zhang Z. et al., 2019). Recently, Li D. et al. (2021) demonstrated that the expression of CSF lncRNA-CCRR was evidently up-regulated in breast cancer metastasis patients, especially in patients with brain metastasis, which provides a direct piece of evidence to demonstrate the dysregulation of CSF lncRNA in brain cancers. On the other hand, Wang M. et al. (2020) found that exosomal LGALS9 in glioblastoma CSF suppressed dendritic cell antigen presentation and cytotoxic T-cell immunity, while blocking the secretion of exosomal LGALS9 could regain sustained tumor antigen-presenting activity of dendritic cells and long-lasting antitumor immunity. Together, CSF lncRNAs have exhibited the significance as promising biomarkers and potential targets for various brain tumors.

## Long Non-Coding RNAs Implications in Various Brain Tumors

### Glioma

**FIGURE 3:**
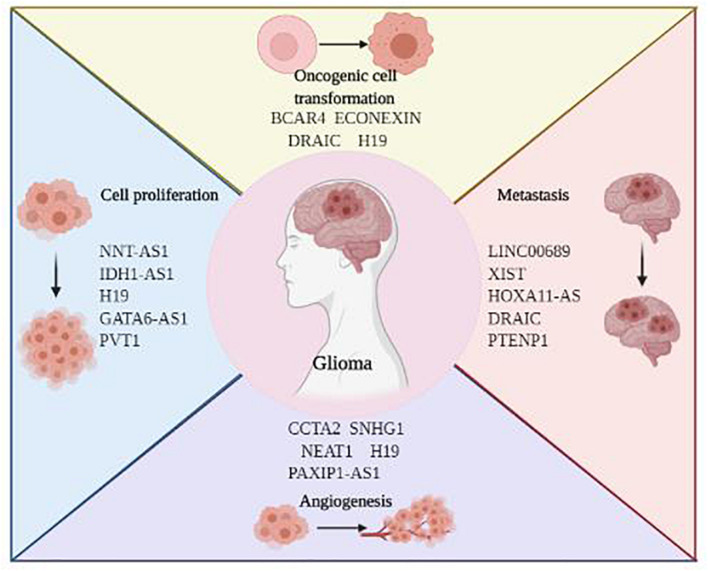
The roles of LncRNAs in glioma development and progression.

As the most prevalent form of primary brain tumors, glioma develops from neural glial cells, mainly star-shaped astrocytes. Based on the classical WHO tumor classification, glioma can be subgrouped into Grade 1–4 (Louis et al., 2016). Despite highly variable histological and genetic characteristics, glioma is notorious for its rapid proliferation, extensive invasion, genetic heterogeneity, and therapeutic resistance. Even given the multiple discipline therapeutics, patients with glioma suffer from the dismal outcomes. Emerging evidence suggests that lncRNAs play a vital role in mediating glioma initiation and progression. Here, we highlighted the current research focusing on *the* implications of lncRNA on glioblastoma and LGG, respectively (Figure 3).

#### Glioblastoma

Glioblastoma (GBM, WHO Grade 4) is the most devastating type of primary brain cancer, accounting for more than half of all primary CNS tumors (Ostrom et al., 2019). In 2016, WHO classified glioblastoma into IDH (isocitrate dehydrogenase) wild-type, IDH mutant, and NOS groups (Louis et al., 2016). In the recently updated WHO classification, glioblastoma is further defined as IDH wild-type adult-type diffuse gliomas (Grade 4), with the iconic molecular profiling including TERT promoter mutation, gain of chromosome 7 and loss of chromosome 10, and EGFR amplification (Louis et al., 2021). Glioblastoma diffusely and rapidly grows to infiltrate the white matter tract and eloquent cortex, making it extremely difficult to achieve early diagnosis and maximal safety resection. Despite the optimal therapeutic approaches combined with surgical resection, targeted radiotherapy, high-dose chemotherapy as well as novel electric field treatment, the median overall survival (OS) of glioblastoma is still less than 21 months, and the 5-year survival rate is between 1–19% (Alexander and Cloughesy, 2017).

In 2019, Yang et al. (2019) reported that glioma stem cell (GSC)-derived lncRNA MALAT1 was transferred to surrounding microglia through exosomal secretion, thereby regulating the inflammatory response through the miR-1295p/HMGB1 axis to affect the secretion of IL-6, IL-8, and TNF-α. This study indicated that exosomal lncRNA played a significant role in maintaining immunosuppressive microenvironment for glioma survival and invasiveness (Yang et al., 2019). Han et al. (2016) demonstrated that lncRNA MALAT1 also mediated anti-glioma effect by suppressing the activation of extracellular regulated protein kinases/mitogen-activated protein kinase (ERK/MAPK) signaling pathway and expression of matrix metalloproteinase 2 (MMP2). Similarly, lncRNA SNHG12, a sponge of miR-129-5p, endows the glioblastoma cells with TMZ resistance by upregulating MAPK1 and activating the MAPK/ERK pathway. Clinically, SNHG12 overexpression was associated with the poor survival of patients treated with TMZ (Lu et al., 2020). Moreover, Tian et al. (2019) found that patients with glioblastoma having high expression of LncRNA AGAP2-AS1 had shorter overall survival time than those with low expression of AGAP2-AS1. The loss-of-function studies showed that downregulation of AGAP2-AS1 depressed cell proliferation, migration, and invasion, and promoted cell apoptosis in glioblastoma. Therefore, AGAP2-AS1 might serve as an oncogenic lncRNA and prognostic biomarker in glioblastoma (Tian et al., 2019). Han et al. (2020) reported that MIR22HG was a critical inducer of the Wnt/β-catenin signaling pathway for glioblastoma, and a specific small-molecule inhibitor, AC1L6JTK, could cause the inhibition of tumor growth *in vivo*. Interestingly, MIR22HG was also enlisted in an immune-related lncRNA signature associated with glioblastoma prognosis, indicating its pathophysiological complexity in this malignancy (Li X. et al., 2021). Together, abundant evidence has revealed that lncRNAs play critical roles in multiple aspects of glioblastoma biology.

#### Low Grade Glioma

In the recently updated WHO classification, the term “anaplastic” was not routinely included; therefore, familiar names like “anaplastic astrocytoma” and “anaplastic oligodendroglioma” were abandoned. Under this circumstance, LGG is now designated as WHO grade 2 astrocytoma and oligodendroglioma, accounting for approximately 5–10% of all CNS tumors (Ostrom et al., 2016). The classical low grade astrocytoma is IDH-mutant, with alternations of ATRX, TP53, and CDKN2A/B. Chromosome 1p19q codeletion is the gold standard characteristics to distinguish oligodendroglioma apart from astrocytoma, along with the alternations of TERT promoter, CIC, FUBP1, and NOTCH1 (Louis et al., 2021). Compared to glioblastoma, LGG has highly variable clinical behavior (Brat et al., 2015). Patients with certain subtype of low grade glioma can survival for decades, while some others progress to secondary glioblastoma within months (Gusyatiner and Hegi, 2018). To improve the diagnostic accuracy and optimize treatment selection, numerous attempts have been made, from the classical histologic characterization to novel molecular profiling, including lncRNA. Nevertheless, considerable controversies remain nowadays. Meanwhile, maximum safety resection might not always be feasible due to the diffuse invasion nature of LGG, and radiotherapy and chemotherapy are alternatively recommended (Franz et al., 2013, 2014).

Growing evidence demonstrates the regulatory role of lncRNA in LGG biology. For example, PR-lncRNA refers to the lncRNAs regulated by P53. *In vitro* experiments of glioma tissues and cell lines revealed that PR-lncRNA expression was negatively correlated with SOX1, SOX2, and SOX9 stem cell factors. Knockdown of SOX abolished the role of PR-lncRNA silencing in glioma cell activity, indicating that the expression and function of PR-lncRNA were significantly altered in LGG formation (Torres-Bayona et al., 2018). LncRNA H19 can enhance tumorigenesis by directly inducing the c-Myc oncogene (Barsyte-Lovejoy et al., 2006). The expression level of H19 was positively correlated with glioma malignancy (Xiao et al., 2020), and H19 could affect the immune infiltration level of glioma through changes in copy number (Yao et al., 2015). As a sponge for miR-675, H19 can regulate the proliferation and migration of glioma cells by producing miR-675 to inhibit the expression of CDK6 (Shi et al., 2014; Li et al., 2016). Moreover, lncRNA LINC00174 is extensively associated with a variety of cancers, including glioblastoma and LGG. In particular, LINC00174 facilitates glycolysis and tumor growth by regulating the miR-152-3p/SLC2A1 axis and regulates the miR-138-5p/SOX9 axis to promote chemotherapeutic resistance to temozolomide in glioma (Shi et al., 2019; Li B. et al., 2020). Therefore, lncRNAs have the potential for diagnostic and prognostic biomarkers in patients with LGG.

### Pituitary Adenoma

Pituitary adenomas are mostly located in the anterior lobe of the pituitary gland, which are usually slow-growing and benign. The symptoms of pituitary adenoma may include headache, optic nerve compression, and endocrine changes, including menopause, gigantism, acromegaly, and Cushing’s disease (Bronstein et al., 2011; Holmes, 2016). In 2018, Zhu et al. (2018) suggested that osteoclast differentiation in bone-invasive pituitary adenomas was directly induced by TNFα, which was further regulated by lncRNA SNHG24. Wu et al. (2018) reported that lncRNA H19 inhibited the phosphorylation of 4E-BP1 by preventing the binding of 4E-BP1 to Raptor. In contrast, the overexpression of H19 significantly inhibited the growth of pituitary tumor cells through cell membrane transport. Zhang et al. further demonstrated that the prognosis of patients with prolactinoma was closely related to the expression level of exosomal H19. In addition, the use of cabergoline could enhance the expression of H19 to exert a synergistic therapeutic effect with exosomal H19, which implies the potential of exosomal H19 in the diagnosis and treatment of pituitary tumors (Zhang Y. et al., 2019). Recently, Mao et al. (2020) reported that lncRNA SNHG6 induced the epithelial-mesenchymal transition (EMT) in pituitary adenomas by inhibiting miR-944. Simultaneously, SNHG6/miR-944/RAB11A axis regulated pituitary adenoma proliferation and invasive behavior (Mao et al., 2020).

### Meningioma

Meningiomas are the most common benign intracranial tumors arising from the arachnoid cells in the meninges, accounting for 38.3% of all CNS tumors and 54.5% of non-malignant CNS tumors (Louis et al., 2016; Ostrom et al., 2020). The overall survival for patients with benign meningiomas is good, whereas the 5-year survival rate for patients with atypical or malignant meningiomas (WHO grade 2 and 3) is less than 60% (Rohringer et al., 1989). Chromosomal abnormalities at 14q32 are commonly implicated in meningioma pathogenesis and progression (Simon et al., 1995; Weber et al., 1997; Martínez-Glez et al., 2010). MEG3, an imprinted gene located at 14q32, can encode non-coding RNAs with antiproliferative functions (Ghafouri-Fard and Taheri, 2019). Ding et al. (2020) confirmed that lncRNA MEG3 mediated the invasive behavior of meningioma cells through the miR-29c/AKAP12 axis. The upregulation in miR-29c levels can eliminate the adverse effects caused by MEG3 expression on the cell cycle, migration, invasion, and proliferation of meningioma cells (Ding et al., 2020). Zhang et al. (2010) also demonstrated that MEG3 mRNA was highly expressed in normal arachnoid cells but was lost in human meningioma cells, and there was a strong association between the loss of MEG3 expression and tumor grade. Additional evidence has shown that MEG3 could induce the expression of P53, which is a tumor suppressor gene in humans; that is, MEG3 overexpression could reduce the proliferation and metastasis of gastric cancer cells (Wei and Wang, 2017). Alternatively, lncRNA SNHG1 is also associated with meningioma progression. Zhao et al. (2019) reported that SNHG1 was overexpressed in meningioma cell lines, of which deficiency restrained cell growth and accelerated apoptosis. Further mechanism experiments demonstrated that SNHG1/miR-556-5p/TCF12 feedback loop promotes proliferation and inhibits apoptosis in meningiomas through the Wnt signaling pathway (Zhang Y. et al., 2020).

### Medulloblastoma

Medulloblastoma is the most common pediatric malignant brain tumor in clinical practice and accounts for 9.2% of pediatric brain tumors (Louis et al., 2007; Millard and De Braganca, 2016; Ostrom et al., 2017). It usually occurs in the lower cerebellar hilum and then metastasizes through CSF circulation (Dufour et al., 2012; Amirjamshidi, 2017). Therefore, CSF cytology has long been one of the routine tests for medulloblastoma. New WHO CNS tumor classification has altered the subgroups of medulloblastomas to mirror new knowledge of their clinical and biological heterogeneity, including 4 principal molecular groups: WNT-activated, sonic hedgehog (SHH)-activated, group 3, and group 4 (Pietsch et al., 2014). SHH is the most common in infants and adults, while other subtypes are common in children (Northcott et al., 2012a, 2017; Taylor et al., 2012). These classifications provide targets for personalized therapy, some of which are currently being tested clinically.

Exploring on the novel molecular biomarkers in medulloblastoma initiation and progression has drawn extensive attention (Ramaswamy and Taylor, 2017). Li B. et al. (2019) reported that lncRNA TP73-AS1 and EIF5A2 were upregulated in medulloblastoma, while miR-494-3p was downregulated. They identify that ELF5A2 is a direct target of miR-494-3p, and knockdown of TP73-AS1 inhibits the proliferation, invasion, and migration of medulloblastoma and promotes cell apoptosis. These findings suggest that lncRNA TP73-AS1 is involved in the development of medulloblastoma as a pro-oncogene (Li B. et al., 2019). Gao et al. (2018a) found that lncRNA LOXL1-AS1 promoted the proliferation and metastasis of medulloblastoma by activating the PI3K/AKT pathway, providing evidence that knockdown of LncRNA LOXL1-AS1 might be a potential therapeutic strategy against medulloblastoma. Moreover, Zhang J. et al. (2020) identified that the expression of lncRNA HOTAIR was higher in medulloblastoma tissues and cell lines than normal samples, which promoted tumor growth, migration, invasion, and EMT by negatively regulating miR-1 and miR-206.

### Primary Central Nervous System Lymphoma

PCNSL refers to the aggressive non-Hodgkin lymphoma in the brain without systemic involvement (Olson et al., 2002; van der Sanden et al., 2002). The most common type of PCNSL is diffuse large B-cell lymphoma (DLBCL), accounting for approximately 2–3% of brain tumors (Langner-Lemercier et al., 2016; Swerdlow et al., 2016; Fox et al., 2019). PCNSL poses an extraordinary challenge to oncologists and neurosurgeons because the impermeability of the BBB hinders the delivery of common chemotherapeutic drugs to the brain (Hanjin et al., 2018). Therefore, intrathecal chemotherapy (injection of chemotherapeutic agents into the CSF via lumbar puncture and delivery of chemotherapeutic agents to the CNS via the cerebrospinal circulation) is reasonable for this malignancy (Cortelazzo et al., 2017).

Recent studies have demonstrated that lncRNAs were widely involved in the biological mechanism of DLBCL by regulating of vital downstream factors through “sponge” intracellular molecules (Huang et al., 2020). For example, lncRNA MALAT1 acts as a ceRNA sponge for miR-195 to stimulate DLBCL cell proliferation and immune escape by activating the immune checkpoint molecules PD-L1 (Wang et al., 2019). LncRNA SNHG14 can act as a ceRNA sponge for miR-5590-3p to upregulate the downstream protein zinc finger E-box binding homeobox 1 (ZEB1). Meanwhile, ZEB1 inversely promotes immune escape of DLBCL cells by transcriptionally activating SNHG14 and PD-L1 (Zhao et al., 2019). SNHG12, another member of the lncRNA SNHG family, can also promote the tumorigenesis of DLCBL by stimulating miR-195 spongiosis (Chen et al., 2020).

### Brain Metastases

Brain metastases refer to the malignant tumors metastasizing to the brain, which are the most common intracranial tumors in adults. Brain metastases may occur up to 10 times more frequently than primary brain tumors (Schouten et al., 2002; Barnholtz-Sloan et al., 2004; Tominaga et al., 2015). Primarily, brain metastases are located in the cerebral hemispheres (80%); the rest were found in the cerebellum and brainstem (Eichler and Loeffler, 2007; Suh et al., 2020). Common cancers causing brain metastases include lung cancer, melanoma, breast cancer, and renal cell carcinoma (Xie et al., 2014). Primary lung cancer is the most common cancer source for brain metastases (Gould, 2018). However, the mechanism underlying the metastatic procedure remains poorly understood. In the cases with multiple metastases, surgery alone usually cannot achieve local control (Mahajan et al., 2017; Churilla et al., 2019). Although whole-brain radiation therapy is acceptable, it is associated with significant cognitive decline (Chang et al., 2009). Similarly, the efficiency of systemic chemotherapy is quite limited, mainly because of the drug resistance and poor penetration through the BBB.

Shen et al. (2015) reported that lncRNA MALAT1 promoted brain metastasis by inducing epithelial-mesenchymal transition in lung cancer, while silencing MALAT1 inhibited highly invasive metastasis cancer cell migration and metastasis by inducing EMT. Furthermore, lncRNA lnc-BM is believed a prognostic indicator for intracranial metastasis patients with breast cancer. Elevated lnc-BM expression promotes STAT3-dependent ICAM1 and CCL2, which mediates communication between breast cancer cells and microenvironment immune cells (Wang et al., 2017). Although the differences in lncRNA expression profiles between primary and metastatic cancer are still controversial, this points to the possibility of distinguishing certain types of metastatic brain tumor with CSF biopsy (Tahira et al., 2011). For instance, Li and colleagues observed the upregulation of lncRNA-CCRR in CSF of metastatic brain tumor from breast cancer.

## The Potential of Cerebrospinal Fluid Long Non-Coding RNAs in the Diagnosis and Treatment of Brain Tumors

### Cerebrospinal Fluid Long Non-coding RNAs as Brain Tumors Diagnostic and Prognostic Biomarkers

Clinical diagnosis of brain tumors depends on the evaluation of symptoms and signs, neuroimaging (such as CT, MRI, and PET-CT), and pathological examination of tissues as the gold standard (Di Lullo and Kriegstein, 2017). In this era of precision medicine, non-invasive neuroimaging can hardly provide the necessary molecular profiling of brain tumors or prognostic information. On the other hand, serial samples of brain tumors are difficult to obtain, and, therefore, tracking tumor progression is complex. Moreover, surgical biopsy of the brain tissue is challenging because of the tumor location and hemorrhagic risk. Alternatively, brain tumor biomarkers acquired from the body fluid, including circulating tumor cells, cell-free DNA, and exosomal ncRNAs, have drawn extensive attention to overcome these limitations (Mattox et al., 2019; Le Rhun et al., 2020).

A cancer biomarker can be a substance naturally produced by a tumor or the body’s unique reaction to the presence of diseases (Ariston Gabriel et al., 2020; Simonato et al., 2021). Regarding brain tumors, the presence of BBB hinders the transport of nucleic acids and proteins and dramatically decreases the concentration of tumor biomarkers in the peripheral blood of patients (Antonetti et al., 2021). In contrast, CSF is more closely associated with brain tissue than serum and can be enriched for tumor specific biomarkers (Killer, 2013; Parnetti et al., 2019; Gaetani et al., 2020). LncRNAs in the CSF, therefore, are supposed to be potential candidates as sensitive and accurate early diagnostic and prognostic tools for various brain tumors (Latowska et al., 2020).

An increasing number of studies have evidenced the key roles of lncRNAs in regulating cell proliferation, apoptosis, GSC self-renewal, differentiation, and response to hypoxic stress of different brain tumors. A previous study by Jing et al. (2016) examined the expression of lncRNA CRNDE in 164 gliomas and neighboring non-tumor tissues. Overexpression of CRNDE was correlated with a higher WHO grade, recurrence, and tumor volume expansion in tumor tissues; therefore, elevated expression of this lncRNA may be considered a new prognostic marker in glioma. Similarly, lncRNA HOTAIR has been widely discussed regarding glioma biology. For instance, Suppressing HOTAIR expression inhibits glioma cell proliferation, migration, and invasion, which involves the PI3K/AKT signaling pathway (Ke et al., 2015). Therefore, this lncRNA could be considered a novel prognostic and diagnostic biomarker for glioblastoma (Tan et al., 2018). Furthermore, the expression of lncRNA miR210HG was substantially upregulated in tumor tissue than adjacent normal tissue. Patients with glioma exhibited substantially higher serum miR210HG level than healthy controls, indicating this lncRNA is a potential diagnostic biomarker for glioma (Min et al., 2016). Considering the complicated biological effects and interactions of lncRNAs, the multiple-lncRNA signatures could be better diagnostic and prognostic indicators rather than single lncRNAs. Zhang et al. (2012) suggested that several lncRNAs could be used to distinguish between the stage and type of glioma. This study indicated that approximately 129 lncRNAs were differentially expressed between gliomas and normal brain tissue, demonstrating the capacity of lncRNAs in tumor stratification. Serial studies focused on medulloblastoma have also shown that a variety of lncRNAs, including CCAT1, CRNDE, Linc-NeD125, and PVT1, were associated with tumor progression (Northcott et al., 2012b; Song et al., 2016; Laneve et al., 2017; Gao et al., 2018b). Among these lncRNAs, Linc-NeD125 was overexpressed in medulloblastoma tissues compared to normal brain tissues; further studies showed that its ectopic expression promoted cell proliferation, migration, and invasion of medulloblastoma cells *in vitro* (Laneve et al., 2017). Interestingly, in a recent report from Li and colleagues, tissue expression and CSF expressions of lncRNA CCRR were both evidently upregulated in breast cancer patients with brain metastases. However, the upregulation of serum lncRNA level was not documented, indicating that CSF lncRNAs might be better biomarkers for intracranial tumors (Li D. et al., 2021). Together, the facts mentioned above indicate that CSF lncRNAs have great potential in diagnosing and predicting brain tumors and provide new approaches for the individualized treatment of patients. More information on lncRNAs as biomarkers for brain tumors is summarized in Table 1.

**TABLE 1:** Prognostic and diagnostic LncRNA biomarkers for brain tumors.

Type of cancer	lncRNAs	Function	References
Glioma	miR210HG	Diagnosis biomarker	Min et al., 2016
	FAM225B	Prognosis biomarker	Li J. et al., 2020
	TP73-AS1	Prognosis biomarker	Mazor et al., 2019
	HOTAIR	Prognosis and diagnosis biomarker	Tan et al., 2018
	HOXA6as	Diagnosis biomarker	Kraus et al., 2015
	EGOT	Diagnosis biomarker	Wu et al., 2017
	GAS5	Diagnosis biomarker	Liu et al., 2018
	FTH1P3	Diagnosis biomarker	Zhang et al., 2018a
	ELF3-AS1	Prognostic biomarker	Mei et al., 2020
Pituitary adenoma	C5orf66-AS1	Diagnosis biomarker	Yu et al., 2017
	H19	Diagnosis biomarker	Zhang Y. et al., 2019
	CCAT2	Diagnosis biomarker	Fu et al., 2018
	RPSAP52	Diagnosis biomarker	D’Angelo et al., 2019
	MEG8	Diagnosis biomarker	Zhu et al., 2021
Medulloblastoma	TP73-AS1	Diagnosis biomarker	Li B. et al., 2019
	LOXL1-AS1	Prognostic biomarker	Gao et al., 2018a; Chen et al., 2019
	lnc-HLX-2-7	Diagnosis biomarker	Katsushima et al., 2021
Meningioma	LINC00702	Diagnosis biomarker	Li T. et al., 2019
	MEG3	Diagnosis biomarker	Ding et al., 2020

Although CSF lncRNAs is difficult to be used as a routine screening nowadays, it has exhibited several advantages. First, serial CSF lncRNAs can be acquired by lumber puncture, to monitor the progression in a micro-invasive and dynamic way. Second, CSF lncRNAs are directly secreted and confined within the CNS to eliminate the systemic factors, which helps us to understand the biology and pathophysiology of brain tumors. Third, multiple lncRNA microarray would further improve diagnostic accuracy such as sensitivity and specificity. Fourth, the lowest MRI resolution ranges in the order of millimeters, whereas the dimensions of the tumor cell are in micrometers. Such disparity in scale may lead to delay in diagnosis, which can be compromised by CSF lncRNA biopsy. Finally, lncRNA can be combined with the current neuroimage, rather than replace it. For instance, Wu et al. (2021) observed that lncRNA CASC19 promoted glioma progression by modulating the miR-454-3p/RAB5A axis, which was associated with unfavorable MRI features. Similarly, lncRNA SAMMSON overexpression help distinguishing patients with glioblastoma from diffuse neurosarcoidosis, which shares quite similar radiological features (Xie et al., 2019).

### Cerebrospinal Fluid Long Non-coding RNA as Brain Tumor Therapeutic Agents

Although conventional strategies for brain tumor treatment have been shown to be promising, it remains a considerable challenge to improve the outcomes of patients. For instance, even given the multidiscipline approaches combined with surgical resection, targeted radiotherapy, high-dose chemotherapy, and novel electric field treatment, the median overall survival for glioblastoma is still less than 21 months (Tan et al., 2020). One of the major obstacles is the poor penetration of BBB. Many attempts have been made to deliver drugs efficiently through BBB (Allhenn et al., 2012), including the intrathecal administration (drug injection to the lumbar arachnoid space) and intraventricular administration (drug injection or infusion into the lateral ventricles of the brain, Calias et al., 2014). Kim et al. (2016) evaluated the efficiency of different anti-miR delivery strategies, including intratumoral, intrathecal, and intraventricular routes, in an orthotopic model of GBM. Intraventricular injection of anti-Let-7 resulted in a significant reduction in target gene expression in the whole tumor, indicating a promising approach for ncRNA therapy in brain tumors (Kim et al., 2016). More recently, Donovan et al. (2020) demonstrated that administration of chimeric antigen receptor T (CAR-T) cells into the CSF could be a highly effective therapy for multiple metastatic mouse models of medulloblastoma and PFA ependymoma. Yang et al. (2019) also reported that intrathecal injection of umbilical cord blood mesenchymal stem cells could improve the pain through lncRNA H19/microRNA-29a-3p/FOS axis. These studies suggest that CSF delivery is practical and promising approach to fight against brain tumors. Specifically, intrathecal and intraventricular administration of lncRNAs packaged by exosomes can be evaluated as novel therapeutics for various brain tumors. For instance, Lai et al. (2014) created a sensitive extracellular vesicle system with high stability and BBB permeability.

Generally, lncRNAs participate in the multiple aspects of tumor biology, such as proliferation, invasion, angiogenesis, treatment resistance, stemness maintenance, and immune suppression. Many ongoing studies, therefore, are designed to identify the lncRNAs with potential anti-tumor characteristics, as summarized in Table 2. For instance, TMZ-based chemotherapy is the fundamental treatment for patients with glioma, especially for malignant glioma. Several lncRNAs have been found to be involved in chemoresistance to TMZ in glioma cells, including lncRNA HOTAIR, H19, and MALAT1 (Jiang et al., 2016; Zhang L. et al., 2020). Recently, Lv et al. (2020) demonstrated that high expression of lncRNA DLEU1 predicted a poor prognosis. Furthermore, silencing lncRNA DLEU1 suppressed TMZ-activated autophagy and promoted the sensitivity of glioma cells to TMZ by triggering apoptosis (Lv et al., 2020). Considering the complicated epigenetic effects and interactions of lncRNAs, single lncRNA can be involved in different tumor biological functions. LncRNA LINC00174 is extensively associated with a variety of cancers, including glioblastoma and LGGs. In particular, LINC00174 facilitates glycolysis and tumor growth by regulating the miR-152-3p/SLC2A1 axis and regulates the miR-138-5p/SOX9 axis to promote chemotherapeutic resistance to temozolomide in glioma (Shi et al., 2019; Li B. et al., 2020). It also acts as an oncogene in glioblastoma via promoting proliferative phenotype (Wang Z. et al., 2020). Besides glioma, lncRNAs can be therapeutic targets in other brain cancers. For instance, knockdown of oncogenic lncRNA CRNDE inhibited tumor development in medulloblastoma cell lines, significantly decreased cell proliferation, and increased apoptosis (Song et al., 2016). Similarly, Xing et al. (2018) indicated that lncRNA LINC00460 promoted MMP-9 expression through targeting miR-539, acting as an oncogenic RNA in the meningioma malignancy and accelerating the proliferation and metastasis of meningioma. In conclusion, these studies suggest CSF lncRNAs have shown great potential as a therapeutic target, although further effort is needed before the clinical application.

**TABLE 2:** LncRNAs involved in the treatment of brain tumor.

Type of cancer	lncRNAs	Function	References
Glioma	MIR22HG	Inhibits glioblastoma progression through suppression of Wnt/β-catenin signaling	Han et al., 2019
	HOX	Inhibits the occurrence and progression of glioma	Yang et al., 2018
	TP73-AS1	Therapeutic target	Mazor et al., 2019
	MALAT1	Knockdown reverses chemoresistance to temozolomide via promoting microRNA-101	Cai et al., 2018
	TUSC7	Inhibits temozolomide resistance by targeting miR-10a	Shang et al., 2018
Pituitary adenoma	H19	Inhibits the growth of pituitary adenoma	Zhang Y. et al., 2019
	SNHG24	Induce osteoclast Differentiation of bone-invasive pituitary Adenomas by regulating TNFα	Zhu et al., 2018
	SNHG6	Induces EMT of pituitary adenoma via suppressing miR-944	Mao et al., 2020
	LINC01116	Boost the progression of pituitary adenoma cells via regulating miR-744-5p/HOXB8 pathway	Huang et al., 2021
	LINC00473	Overexpress in IPA and can promote PA cell proliferation	Li J. et al., 2021
	C5orf66-AS1	Plays an anticancer role and significantly Inhibits cell activity and invasiveness	Yu et al., 2017
	MEG3	As a tumor suppressor	Chunharojrith et al., 2015
Medulloblastoma	CCAT1	Promotion of cell proliferation and metastasis	Gao et al., 2018b
	NKX2-2AS	Suppression of cell proliferation Migration and invasion	Zhang et al., 2018b
	linc-NeD125	Ectopic expression of linc-NeD125 in invasive MB cells attenuated their proliferation, migration, and invasion	Laneve et al., 2017
	SPRY4-IT1	Promotion of cell proliferation and migration and invasion	Zhang et al., 2018b

## Conclusion and Prospects

Brain tumors directly threaten the cognition, behavior, and neurologic functions of human beings. While considerable advancements in fundamental scientific research and clinical practice have shed light on brain tumor pathophysiology in the past decades, challenges remain in precise and early diagnosis. LncRNAs exert critical regulatory efforts in the development and progression of different brain tumors, including glioma, meningioma, pituitary adenoma, medulloblastoma, PCNSL, and brain metastasis. Compared to the other ncRNAs, such as miRNAs and circRNAs, the regulatory mechanisms of lncRNAs seems to be more complicated. For instance, miRNAs are small ncRNAs consisting of approximately 21–25 nucleotides, which act as regulators of gene expression by complementary binding of the 3′ untranslated regions (UTR) of targeted mRNAs, thus reducing the mRNA stability or modulating gene translation. On the contrary, lncRNA might exert epigenetic functions in a more comprehensive and complex manner (as shown in Figure 2), including transcriptional regulation of genes, acting as small RNA precursors and miRNA sponges, protein localization alternation, and production of endogenous siRNAs. Also, the interactions between these ncRNAs have been described, which construct a regulatory network of brain cancer. CSF biopsy represents a novel approach to monitor the pathophysiology of brain tumors in an efficient, mini-invasive, and continuous manner. Moreover, intrathecal or intraventricular administration has been demonstrated to deliver multiple drugs and therapeutic agents efficiently through BBB. As we reviewed, CSF lncRNAs provide great promises for clinical applications, including the diagnosis and treatment of brain tumors.

Though promising, several challenges remain to be addressed. First, the trace amount of lncRNAs in the CSF brings considerable difficulties to detection and diagnosis. Nowadays, high-throughput RNA-seq technology develops rapidly, making it possible to simultaneously detect multiple tumor-specific lncRNAs to balance the sensitivity and accuracy of early diagnostics. In fact, numerous bioinformatics-based lncRNA signatures have been established with diagnostic and predictive potential. Second, the bioactivity of certain lncRNA needs to be fully elucidated due to its epigenetic effort before the clinical application. Third, directly targeting CSF lncRNAs is challenging, or even useless, because these lncRNAs are dominantly released to the CSF by tumor cells as biomarkers. To exert maximal therapeutics effort, intrathecal or intraventricular administration of therapeutic agents, such as small interfering RNA (siRNA), antisense oligonucleotide (ASO), small molecule inhibitors, or even exosome-sealed ncRNAs, should be considered to reduce the expression of tissue lncRNAs or inhibit their functions within the tumor microenvironment. Finally, CSF lncRNAs cannot be the only answer for the diagnostics and treatment of brain tumors. Synergy between lncRNAs and other oncogenic regulators has been primarily documented (Parasramka et al., 2017; Wu et al., 2020). With progressively better understanding of lncRNA regulatory mechanisms, we believe CSF lncRNAs combined with others wild become increasingly valuable agents in diagnosing and treating various brain tumors.

## Author Contributions

KX and SX conceived the structure of the manuscript and revised the manuscript. KX, XJ, and AAG designed and drafted the manuscript. XJ, YW, XL, and SX discussed and revised the manuscript. All authors read and approved the final manuscript.

## Conflict of Interest

The authors declare that the research was conducted in the absence of any commercial or financial relationships that could be construed as a potential conflict of interest.

## Publisher’s Note

All claims expressed in this article are solely those of the authors and do not necessarily represent those of their affiliated organizations, or those of the publisher, the editors and the reviewers. Any product that may be evaluated in this article, or claim that may be made by its manufacturer, is not guaranteed or endorsed by the publisher.

## Abbreviations

lncRNA: Long non-coding RNA
CSF: Cerebrospinal fluid
CT: Computed tomography
MRI: Magnetic resonance imaging
PET: Positron emission tomography
GBM: Glioblastoma
BBB: Blood-brain barrier
CNS: Central nervous system
miRNA: microRNA
EMT: Epithelial-to-mesenchymal transition
IDH: Isocitrate dehydrogenase
HGG: High-grade glioma
EGFR: Epidermal growth factor receptor
TERT: Telomerase reverse transcriptase
ATRX: A-thalassemiamental retardation syndrome X
GSC: Glioma stem cell
LGG: Low-grade glioma
LPS: Lipopolysaccharide
MMP: Matrix metalloproteinase
TMZ: Temozolomide
PCNSL: primary CNS lymphoma
DLBCL: Diffuse large B-cell lymphoma
ZEB1: Zinc finger E-box binding homeobox 1
UTR: Untranslated regions
siRNA: Small interfering RNA
ASO: Antisense oligonucleotide
ncRNA: non-coding RNA
WHO: World health organization.
